# Chronic Noise Exposure and Risk of Dementia: A Systematic Review and Dose-Response Meta-Analysis

**DOI:** 10.3389/fpubh.2022.832881

**Published:** 2022-06-20

**Authors:** Linghao Meng, Yang Zhang, Shushan Zhang, Fugui Jiang, Leihao Sha, Yajia Lan, Lei Huang

**Affiliations:** ^1^Department of Urology, West China School of Medicine/West China Hospital, Sichuan University, Chengdu, China; ^2^Cochrane China Center, Chinese Evidence-Based Medicine Center, West China Hospital, Sichuan University, Chengdu, China; ^3^Department of Periodical Press and National Clinical Research Center for Geriatrics, West China Hospital, Sichuan University, Chengdu, China; ^4^Department of Neurology, Affiliated Hospital of North Sichuan Medical College, Nanchong, China; ^5^Sichuan Provincial Center for Mental Health, Sichuan Academy of Medical Sciences & Sichuan Provincial People's Hospital, Chengdu, China; ^6^Department of Neurology, West China Hospital, Sichuan University, Chengdu, China; ^7^Department of Environmental Health and Occupational Medicine, West China School of Public Health and West China Fourth Hospital, Sichuan University, Chengdu, China; ^8^Department of Occupational Hazard Assessment, West China School of Public Health and West China Fourth Hospital, Sichuan University, Chengdu, China

**Keywords:** noise exposure, dementia, mild cognitive impairment, Alzheimer's disease, dose-response meta-analysis

## Abstract

**Objective:**

Evidence is scarce about the effect of noise exposure on the risk of dementia. We conducted a systematic review and dose-response meta-analysis, aiming to explore the association between noise exposure and the risk of dementia.

**Methods:**

We searched PubMed, EMBASE and the Cochrane Library to collect studies on chronic noise exposure and the risk of dementia from database inception to September 18, 2021 without language limitations. Two authors independently screened the literature, extracted data and assessed the risk of bias of the included studies. A dose-response meta-analysis and subgroup analysis were then conducted to detect the association between noise exposure and the risk of dementia by using Stata 14.0 software. This study is registered on PROSPERO (CRD42021249243).

**Results:**

A total of 11 studies were eligible for qualitative synthesis, and nine were eligible for quantitative data synthesis. All of them showed moderate to high quality scores in the assessment of risk of bias. We found a positive linear association between the noise increment and dementia risk (*R*^2^ = 0.58). When noise exposure increased 57 dB, the RR of dementia was 1.47 (95% CI: 1.21–1.78). From the outcome subgroup of AD, AD and dementia, VaD and NAD, we also found a positive association (*R*^2^ = 0.68, 0.68, 0.58, respectively). When noise exposure increased by 25 dB, the RRs were 1.18 (95% CI: 1.14–1.23), 1.19 (95% CI: 1.14–1.23) and 1.17 (95% CI: 1.06–1.30), respectively. We found a nonlinear association between the noise increment and dementia risk when only cohort studies were included (*R*^2^ = 0.58). When noise exposure increased by 25 dB, the RR of dementia was 1.16 (95% CI: 1.12–1.20). From the subgroup of AD, AD and dementia, VaD and NAD of cohort studies, the regression curve showed a nonlinear positive association (*R*^2^ = 0.74, 0.71, 0.43, respectively). When noise exposure increased by 25 dB, the RRs were 1.17 (95% CI: 1.12–1.21), 1.17 (95% CI: 1.12–1.22) and 1.13 (95% CI: 0.99–1.28), respectively.

**Conclusion:**

Based on the current evidence, exposure to noise may be a specific risk factor for dementia. To better prevent dementia, more rigorously designed studies are needed to explore the etiological mechanism of noise and dementia.

## Introduction

Dementia is a kind of disease characterized by severe cognitive impairment, including several subtypes, such as Alzheimer's disease (AD), cerebrovascular disease, Lewy body dementia, and Huntington's disease (1). Alzheimer's disease contributes the majority of patients with dementia, accounting for approximately 60~80% of cases (2). Studies have shown that there were approximately 46 million people diagnosed with AD globally in 2015, and the number may reach 115.4 million by 2050 (3). The prevalence of dementia is estimated to be 7% in people above the age of 65, and the rate rises exponentially with age (1, 4). Tau and β-amyloid (Aβ) deposition are considered to be the possible pathological mechanisms of dementia, AD and cognitive impairment (5, 6). However, the cause of dementia has not been expounded completely. Hypertension, diabetes, high body mass index (BMI), smoking, and air pollution are thought to be risk factors for dementia thus far (1, 7). Mild cognitive impairment (MCI) is a state between normal cognition and dementia that is considered a premonitory symptom of dementia with preserved basic cognitive abilities (8). MCI may convert into dementia when specific diagnostic criteria are used or exposed to more risk factors (8, 9). Dementia or MCI can be diagnosed mainly by history-taking, neurological examination and imaging examination (4, 10). However, there was no specific therapy for any subtype of dementia or MCI. Drugs such as cholinesterase inhibitors, N-methyl-D-aspartic acid (NMDA) receptor antagonists, selective serotonin reuptake inhibitor (SSRI) antidepressants and rehabilitation training are only used for symptomatic and supportive treatments (11–13).

With the development of modernization and industrialization, noise is ubiquitous in life. The World Health Organization (WHO) estimated that 1~1.6 million disability-adjusted life years (DALYs) are lost each year due to noise exposure in Western European countries (14). Increasing evidence has shown that noise is associated with many diseases, such as ischemic heart disease (IHD), stroke, metabolic disorders and cognitive dysfunction (15, 16). Recent studies believe that noise may increase the risk of dementia. Animal experiments have shown that environmental noise exposure can influence cognitive performance, along with changes in Tau and β-amyloid (Aβ) at the same time (17, 18). Although it is not clear completely how noise results in dementia pathologically, some papers have reported varied relationships between them. Linares et al. (19) found that short-term exposure to noise may be associated with hospital admission for dementia. Chen et al. (20) found that roads closer to heavy traffic can increase the incidence of dementia, which may be the effect of noise and air pollution. However, Andersson et al. (21) conducted a cohort study in 2018 and reported no association with traffic noise and AD or vascular dementia (VaD). There was not enough evidence to clarify the relationship between them. Several recent reviews also suggested an ambiguous link between noise and dementia (22, 23). Studies have proposed several hypotheses about the pathogenesis mechanism of noise and dementia. Experimental evidence has shown that noise may lead to dementia by neurovascular, neuroendocrine or oxidative stress factors (7, 15, 24, 25). In addition, studies have reported an association between hearing loss and dementia but have not explained the link between hearing loss and noise (26, 27).

A systematic review conducted in 2020 summarized seven studies related to traffic noise with dementia, including Parkinson's disease, dementia, Alzheimer's disease, and hospitalizations for dementia-related illnesses (28). However, this review only focused on traffic noise and did not systematically report traffic noise and the risk of dementia. Furthermore, two reviews conducted in 2018 by the WHO focused on the effect of environmental noise on wellbeing, quality of life and cognitive impairment in multiple dimensions (14, 29). Hegewald et al. (30) carried out a meta-analysis of traffic noise and mental health (including depression, anxiety, dementia and AD) but did not perform a quantitative synthesis of dementia and AD due to different outcomes. Our research team previously summarized the experimental and epidemiological studies of the relationship between chronic noise exposure, cognitive impairment and degenerative dementia but did not conduct a systematic review (31). Some new evidence of dementia emerged after the retrieval time of the studies above so that more evidence can be included and synthesized. Thus, given that there was no quantitative meta-analysis before, in this study, we reviewed existing studies to conduct a systematic review and dose-response meta-analysis, aiming to summarize the evidence regarding the risk of dementia when exposed to chronic noise.

## Materials and Methods

We conducted this systematic review following the Preferred Reporting Items for Systematic Reviews and Meta-analyses (PRISMA) guidelines (32). This systematic review and meta-analysis was registered with PROSPERO (CRD42021249243).

### Search Strategy

Two authors (LM and YZ) independently searched the PubMed, EMBASE and Cochrane Library databases to collect observational studies on chronic noise exposure and the risk of dementia. We searched all studies without language restriction from inception to 18 September 2021. We used MeSH and free text terms to identify the relevant literature. The detailed search strategy of each database is attached in the Supplementary Material. An additional search was performed among the references of the included studies to identify potentially eligible studies. We also manually searched the references of published reviews to collect additional relevant studies.

### Inclusion and Exclusion Criteria

We used a population-intervention-comparator-outcomes-study design (PICOS) to identify the inclusion criteria. (1) Participants: People who were not diagnosed with any cognitive dysfunction (including dementia, Alzheimer's disease or MCI) without age limitations were included in the cohort study; People in case group were diagnosed with any cognitive dysfunction (including dementia, Alzheimer's disease or MCI), and the control group were not diagnosed with any cognitive dysfunction in case-control study. (2) Exposure/Control: Exposure group exposed to chronic noise, including traffic noise, community noise and occupational noise. The control group was not exposed to chronic noise. (3) Outcome: Association between chronic noise exposure and dementia. (4) Study design: Cohort studies, case-control studies or cross-sectional studies were included in this study. Because of the restrictions of research topics and ethics, no randomized controlled trials (RCTs) can be included.

The exclusion criteria for this study were as follows: (1) cohort studies included people diagnosed with any cognitive dysfunction before baseline; case-control studies included people with major psychiatric disorders or significant disease that could interfere with cognition; (2) studies that explored the association between hearing loss and dementia, cognitive dysfunction or AD but did not search for the relationship with hearing loss and noise exposure; (3) studies that could not extract data; (4) *in vitro* or animal experiments; (5) duplicate data; (6) reviews, letters, case reports, protocols, conference abstracts and any article without full text; and (7) studies were not published in English.

### Data Extraction

Two authors (LM and YZ) independently selected the studies by title, abstract and full text. Disagreements were resolved by discussion or consultation with a third reviewer (LH). We extracted the data from each retained study by using a unified information extraction table. The extracted terms included first author, study country/region, publication year, study design, inclusion number, analysis number, sex, age, follow-up periods, noise (sources, level and assessment method), dementia (type and assessment method), number of patients, adjusted covariates and risk estimate indicators, including the relative risk (RR), hazard ratio (HR), odds ratio (OR) and regression coefficient (β). If the risk indicators in each study were adjusted by covariates, we extracted all the adjusted risk indicators. The main adjusted factors included (1) baseline information: age, sex, body mass index; (2) socioeconomic status: education, household income occupational environment; (3) disease and lifestyle factors: stroke, cardiovascular disease, ApoE4 level, smoking, alcohol consumption, physical activity; and (4) other exposure: NO_x_, PM_2.5_, PM_10_. For the studies that had missing main data, we asked the authors for the full data by email. One author extracted the data, and another author checked the accuracy. The questions were solved through the examination of the original articles and discussion.

### Risk of Bias Assessment

Two authors (LM and YZ) independently assessed the risk of bias using the Newcastle-Ottawa scale (NOS) for cohort and case-control studies and Agency for Healthcare Research and Quality (AHRQ) recommended items for cross-sectional studies. Disagreements were resolved by discussion or consultation with a third reviewer (LH). There is no recognized tool to assess the risk of bias in observational studies. The NOS is the most commonly used scale for cohort studies or case-control studies and is a semiquantitative system (33, 34). The assessment of bias of cohort studies and case-control studies included eight domains. And the details and scales of evaluation can be available in reference (30, 35). The Agency for Healthcare Research and Quality (AHRQ) recommended using 11 terms to assess the risk of bias of cross-sectional studies (https://www.ahrq.gov/). The details of the assessment method are available in reference (36).

### Statistical Analysis

We used Stata 14.0 to perform the meta-analysis. Aiming to clearly research the relationship between noise exposure and dementia, we conducted a dose-response analysis between the dose of noise exposure and the risk of dementia (37–39). L_den_ is an annual long-term average A-weighted equivalent continuous noise level integrating compound metrics (40, 41). Given that the retained studies used different noise assessment methods to evaluate the exposure quantitatively, we converted the different noise metrics into the most commonly used method (L_den_) (15, 16). The detailed conversion methods were available in the study raised by Brink et al. (40). The median dose of the category groups was considered the corresponding dose. When the boundary of the category was open, the median dose of this category was set at the lower boundary multiplied by 1.5 in the highest category, and the dose was set as zero in the lowest category (38). A one stage robust error meta-regression method (REMR) was utilized to analysis the dose-response associations. In this study, restricted cubic spline (RCS) with three knots was applied to model potentially nonlinear associations, and centering was done to eliminate the effects of inconsistency in reference group by subtracting the reference dose from the non-reference dose for each reported non-reference effect (42). *R*^2^ was used to measure the overall fit of the regression equation, and slope equality test was applied to test for nonlinear trends (42–45). Subgroup analysis was performed by different categories of dementia. Publication bias and sensitivity analysis were not performed on analyses with <10 studies due to the low sensitivity of qualitative and quantitative tests.

## Results

### Search Results and Study Characteristics

**Figure 1:**
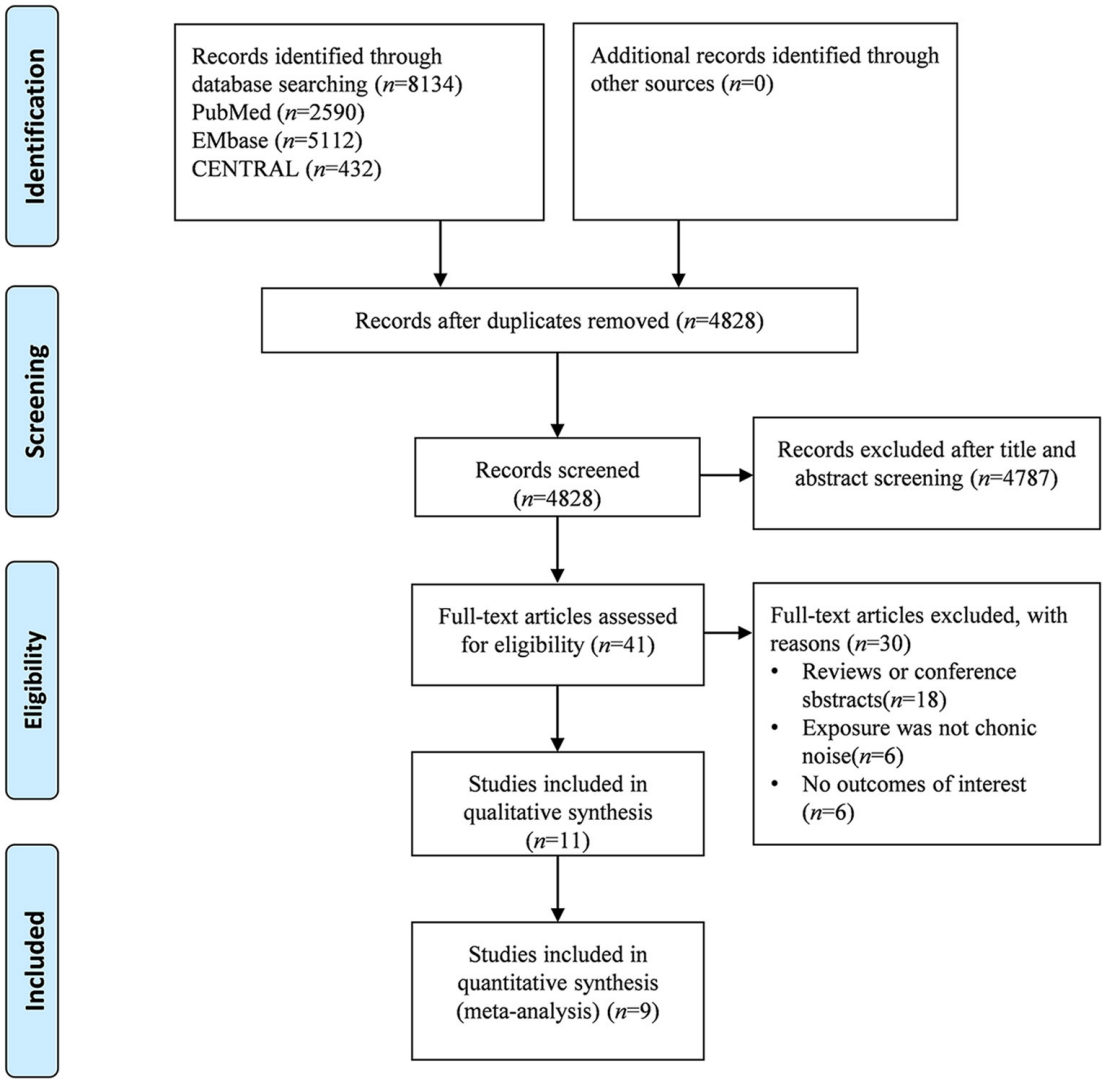
Flowchart of literature screening.

The study search and selection process is shown in Figure 1. From the three databases, we identified 4,828 publications after removing duplications. After title and abstract retrieval, 41 studies were included in the full text screening. Of the potentially eligible publications after full text screening, we excluded 18 studies because they were just reviews or systematic reviews; 6 studies because the exposure was not noise; and six studies had no outcomes of interest. Finally, 11 studies (21, 41, 46–53) were eligible for qualitative synthesis, and nine studies (21, 41, 46, 47, 49, 51–53) were eligible for quantitative data synthesis.

The baseline information of the included studies is shown in Table 1. The risk estimate between noise exposure and dementia outcomes from each reviewed study is attached in the Supplementary Material. Among the 11 eligible studies, five studies were from North America, including two studies from Canada and three from the USA. The other five studies were from Western/Northern Europe (Sweden, Germany, The UK, Danmark and Spain). Eight studies were cohort studies (21, 46, 47, 49, 50, 52, 53), two were cross-sectional studies (48, 51) and one (41) included cohort studies and case-control studies. The noise sources in the 11 studies included traffic noise and community noise. Most of the studies used L_den_, L_nihgt_ or L_eq_ indicators to describe noise magnitude. The studies used different outcomes to describe cognitive impairment, including dementia, vascular dementia (VaD), Alzheimer's disease, mild cognitive impairment (MCI) and non-Alzheimer's dementia (NAD). In the included studies, two studies were based on the same first author and study population (53), in which the data were extracted. In addition, some studies reported multiple different indicators in one study, and we also extracted all the data severely.

**Table 1:** Characteristics of the included studies.

**First author, year**	**Country/ Region**	**Study design**	**Total sample/ Analysis sample**	**Sex (male/ female)**	**Age (range/ mean)**	**Follow-up time (years)**	**Noise type**	**Noise assessment**	**Noise value (dB)**	**Types of dementia**	**Outcome assessment**	Quality assessment (NOS/AHRQ)*
Andersson et al. (21)	Umeå, Sweden	cohort study	1721/1721	985/736	55–85/68.5 ± 9.4	7	traffic noise	Umeå Municipality Noise Survey/Leq.24 h	<55; ≥ 55	dementia	NINCDS–ADRDA^a^	7
Tyas et al. (50)	Manitoba, Canada	cohort study	1355/694	261/433	65–93/74.0 ± 5.8	5	community noise	scale	NA	AD	NINCDS–ADRDA	7
Weuve et al. (52)	Chicago, USA	cohort study	7909/5227	1986/3241	NA/73.7 ± 6.9	4.1	community noise	universal kriging model developed for the Chicago area/Leq 10:00–16:00	range 51.1–78.2	AD; MCI	NINCDS–ADRDA	8
Yuchi et al. (41)	Metro Vancouver, Canada	case–control study	13498/13498	NA	45–84/NA	5	community noise	Lden	case range 33.0–86.5; control range 4.4–92.4	AD	hospital records	7
		cohort study	633949/ 633949	300197/ 333752	45–83/NA			Lden	case range 44.5–77.2; control range 32.2–85.8	NAD	hospital records	9
Fuks et al. (49)	North Rhine–Westphalia, Germany	cohort study	834/288	0/288	67–80/74.5± 2.2	25	community noise	Lden	mean 55.9 ± 7.7	MCI	CERAD Neuropsychological Assessment Battery^b^	7
								Lnight	mean 47.2 ± 7.4	MCI	CERAD Neuropsychological Assessment Battery	
							traffic noise	ICBEN Daytime	NA	MCI	CERAD Neuropsychological Assessment Battery	
								ICBEN Night	NA	MCI	CERAD Neuropsychological Assessment Battery	
Yu et al. (53)	California Sacramento Valley, USA	cohort study	1789/1612	680/932	>60/70.2 ±6.8	6.5	traffic noise	Leq; 24-h Noise/Nighttime Noise	mean Lden=68.5 ± 8.9 Lnight=60.4 ± 8.9	MCI	3 MSE/SEVLT^c^	7
Yu et al. (53)	California Sacramento Valley, USA	cohort study	1789/1612	680/932	>60/70.2 ±6.8	6.5	traffic noise	Leq; 24-h Noise	mean Lden=68.5 ± 8.9	MCI	3 MSE/SEVLT	7
Tzivian et al. (51)	Ruhr, Germany	cross–sectional study	4157/2050	1007/1043	NA/64.1 ± 7.7	NA	traffic noise	Lden; Lnight	mean Lden=53.74 ± 9.49 Lnight=44.88 ± 9.17	MCI	International Working Group on MCI criteria	10
Carey et al. (47)	London, England	cohort study	139718/ 130978	65130/ 65848	50–79/NA	6.9	traffic noise	TRAffic Noise EXposure (TRANEX) model/ Lnight	mean 52.1 ± 4.6	AD; VaD	NA	8
Crous-Bou et al. (48)	Barcelona, Spain	cross– sectional study	228/288	136/92	NA/57.7 ±7.6	NA	traffic noise	Lden; Lnight	mean 67.7 ± 5.77	AD	MRI^d^	6
Cantuaria et al. (46)	Danmark	cohort study	1938994/ 1938994	907991/ 1031003	≥60/NA	8.5	road traffic noise	Lden max	mean 55.3	AD; VaD	clinical diagnosis	8
							railway traffic noise	Lden max	mean 44.3	AD; VaD	clinical diagnosis	
							road traffic noise	Lden min	mean 51.6	AD; VaD	clinical diagnosis	
							railway traffic noise	Lden min	mean 44.7	AD; VaD	clinical diagnosis	

^*^, Newcastle–Ottawa Scale (NOS) for cohort and case–control studies and Agency for Healthcare Research and Quality (AHRQ) recommended items for cross–sectional studies. ^a^, National Institute of Neurological and Communicative Diseases and Stroke/Alzheimer's Disease and Related Disorders Association (NINCDS–ADRDA) criteria. ^b^, Consortium to Establish a Registry on Alzheimer's Disease (CERAD) Neuropsychological Assessment Battery. ^c^, Modified Mini–Mental State Examination (3 MSE) and Spanish English Verbal Learning Test (SEVLT). ^d^, Magnetic Resonance Imaging.

### Risk of Bias Assessment

With regard to different types of studies, we used different assessment methods to evaluate the risk of bias. The quality scores are presented in Table 1, and evaluation details are provided in the Supplementary Material. All the included cohort studies and case-control studies showed high quality scores. One cross-sectional study was rated as high quality (51), and the other was rated as moderate quality (48).

### Dose-Response Meta-Analysis

**Figure 2:**
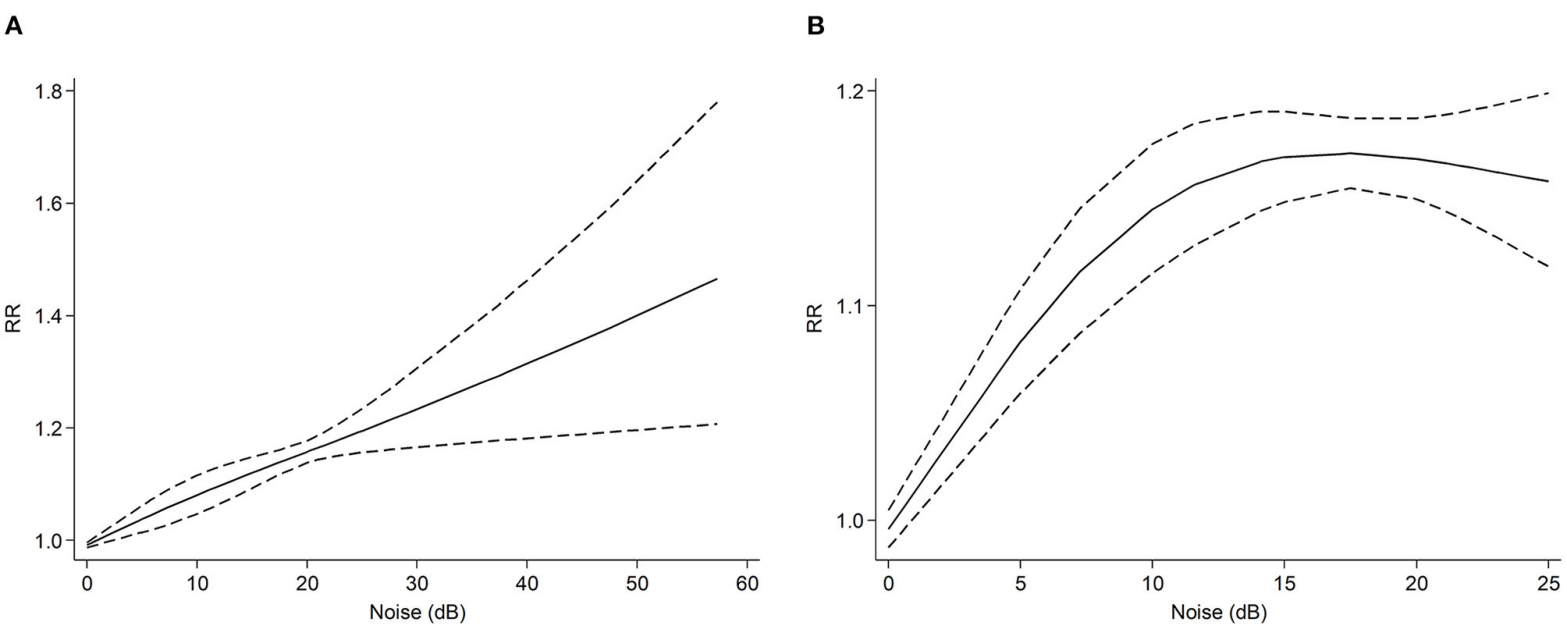
Dose-response meta-analysis between noise exposure and risk of all kinds of dementia. The horizontal axis represents the noise increment, and the vertical axis represents the relative risk of noise increase to dementia. The two dashed lines represent the upper and lower limits of the 95% confidence interval for relative risk. **(A)** dose-response analysis of all studies. **(B)** dose-response analysis of cohort studies.

A total of nine studies (21, 41, 46, 47, 49, 51–53) were included in the dose-response meta-analysis, which contained a population of 2,728,317. Heterogeneity analysis results showed that there was heterogeneity among the studies (*I*^2^ = 78.7%, *P* < 0.001). The noise range of the studies we analyzed was from 22.5 dB to 100.3 dB. From the dose-response analysis of all studies (Figure 2A), we found a positive linear association between the noise increment and dementia risk (*R*^2^ = 0.58, slope test: *F*= 1.53, *P*= 0.223). When noise exposure increased 57 dB, the RR of dementia was 1.47 (95% CI: 1.21–1.78). Seven cohort studies including 2,078,820 participants reported chronic noise exposure and the risk of dementia (21, 46, 47, 49, 52, 53). Heterogeneity analysis results showed that there was heterogeneity among the studies (*I*^2^ = 50.0%, *P* < 0.001). When only these cohort studies were included (Figure 2B), we also found a non-linear association between noise increment and the risk of dementia (*R*^2^ = 0.58, slope test: *F* = 39.38, *P* < 0.001). When noise exposure increased by 25 dB, the RR of dementia was 1.16 (95% CI: 1.12–1.20).

### Subgroup Analysis

**Figure 3:**
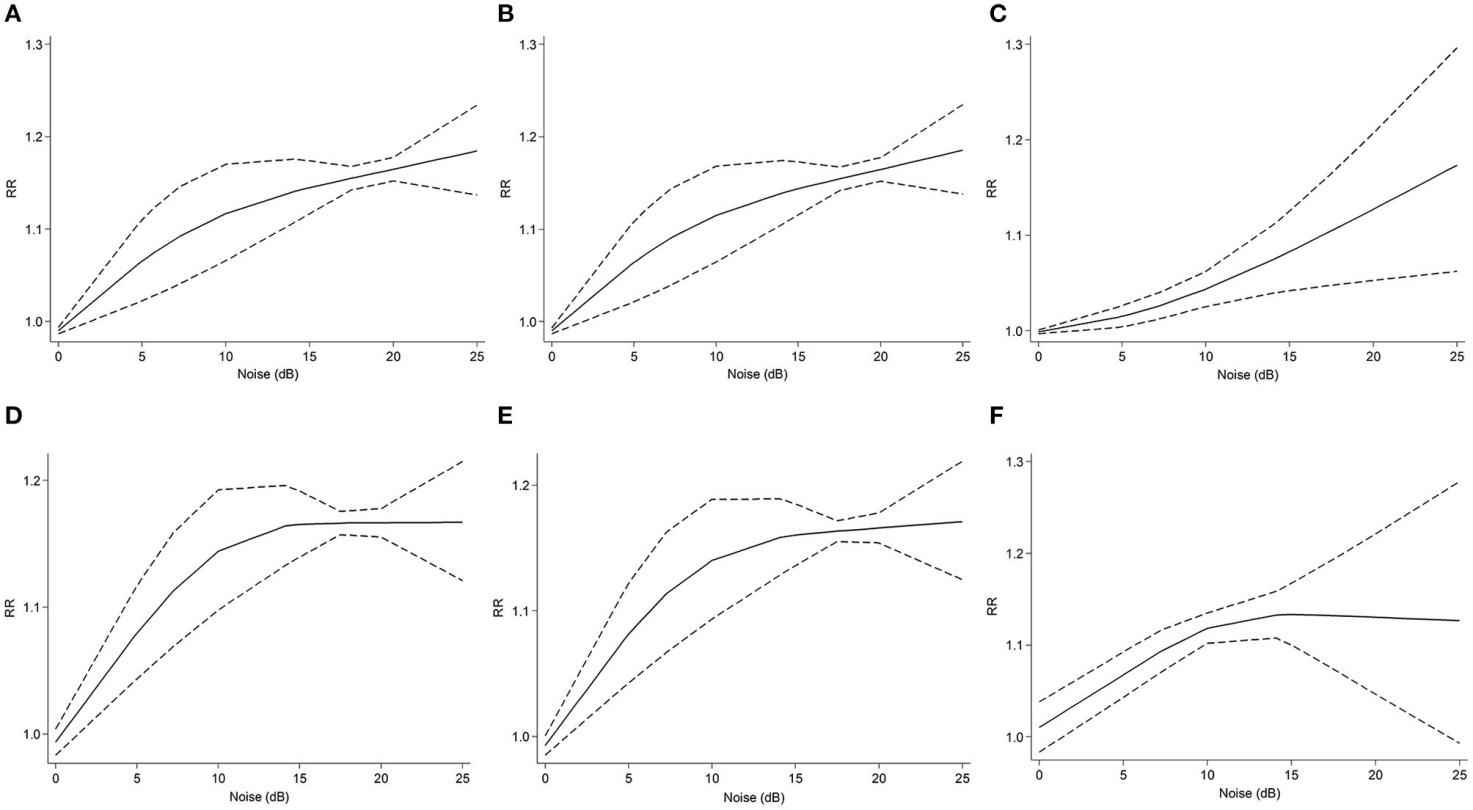
Dose-response meta-analysis between noise exposure and risk of different types of dementia. The horizontal axis represents the noise increment, and the vertical axis represents the relative risk of noise increase to dementia. The two dashed lines represent the upper and lower limits of the 95% confidence interval for relative risk. **(A)** dose-response analysis of noise increment and AD risk in all studies. **(B)** dose-response analysis of noise increment and risk of AD and dementia in all studies. **(C)** dose-response analysis of noise increment and risk of VaD and NAD in all studies. **(D)** dose-response analysis of noise increment and AD risk in cohort studies. **(E)** dose-response analysis of noise increment and risk of AD and dementia in cohort. **(F)** dose-response analysis of noise increment and risk of VaD and NAD in cohort studies.

Five studies (21, 41, 46, 47, 52) reported noise exposure and the risk of AD (*I*^2^ = 78.1%, *P* < 0.001). The outcome showed a nonlinear positive correlation (*R*^2^ = 0.68, slope test: *F* = 5.11, *P* = 0.047). When the noise increment was within 10 dB, as the noise increased, the relative risk of AD rose steeply (Figure 3A). In contrast, the curve rises slowly in the range of more than 10 dB. When noise exposure increased by 25 dB, the RR was 1.18 (95% CI: 1.14–1.23).

From the outcome subgroup analysis of AD and dementia, we discovered a nonlinear positive association (*R*^2^ = 0.67, slope test: *F* = 4.90, *P* = 0.049). Five studies (21, 41, 46, 47, 52) were included in this subgroup (*I*^2^ = 75.0%, *P* < 0.001). The characteristics of the dose-response analysis were similar to those of the AD subgroup (Figure 3B). When noise exposure increased by 25 dB, the RR was 1.19 (95% CI: 1.14–1.23). Regarding the subgroup analysis of VaD and NAD, four studies (21, 41, 46, 47) were included (*I*^2 =^68.5%, *P* < 0.001). The dose-response analysis presented a nonlinear correlation (*R*^2^ = 0.58, slope test: *F* = 0.21, *P* = 0.660), with a 25 dB noise increment RR of 1.17 (95% CI: 1.06-1.30) (Figure 3C).

We also conducted a subgroup analysis of the cohort studies. Four cohort studies (21, 46, 47, 52) were included in the AD subgroup (*I*^2 =^67.1%, *P* < 0.01). The dose-response analysis showed a nonlinear association (*R*^2 =^ 0.74, slope test: *F*=19.72, *P*=0.004) (Figure 3D). When noise increased by 25 dB, the RR was 1.17 (95% CI: 1.12–1.21). In the four cohort studies (21, 46, 47, 52) of the subgroup of AD and dementia (*I*^2^ = 63.0%, *P* < 0.01), the dose–response analysis showed a nonlinear association (*R*^2^ = 0.71, slope test: *F* = 13.69, *P* = 0.008) (Figure 3E). When noise increased by 25 dB, the RR was 1.17 (95% CI: 1.12–1.22). Three cohort studies (21, 46, 47) were included in the subgroup of VaD and NAD (*I*^2^ = 8.9%, *P* = 0.35). There was also a nonlinear association between noise exposure and outcomes (*R*^2 =^ 0.43, slope test: *F* = 39.48, *P* = 0.008) (Figure 3F). When noise exposure increased by 25 dB, the RR was 1.13 (95% CI: 0.99–1.28).

The above three subgroups of cohort studies presented similar dose response analysis characteristics. When the noise exposure increment was under 15 dB, the RR increased steeply as the noise increased. The curve began to level off in the range of more than 15 dB.

### Sensitivity Analysis and Publication Bias

Considering that there were fewer than 10 eligible studies, sensitivity analysis and Begg's and Egger's tests for publication bias were not used for the meta-analysis due to the low efficiency of qualitative and quantitative tests.

## Discussion

From the meta-analysis of the nine observational studies, we found a significant positive association between the noise exposure increment and the risk of dementia, which was identical to previous experimental studies. For the subgroup analysis, the groups were divided based on the different subtypes of dementia to explore the source of heterogeneity. The AD group, the AD and dementia group, and the VaD and NAD group showed similar positive associations between noise exposure and the risk of outcomes. The subgroup analysis of cohort studies presented similar dose-response analysis characteristics. When the noise exposure increment was under 15 dB, the RR increased steeply as the noise increased.

In this study, we used REMR method to conduct the dose-response meta-analysis. The REMR method is a special case of the one-stage generalized least-squares trend (GLST) approach, which may have better error estimation and a better visual fit to the data than GLST model (42). The execution of the model need not extract the covariation from the data. It has been reported that the model can be an alternative method for the synthesis of dose-response data from different studies when there is lack of independence in estimates from the same study (42, 45).

From our study, we found associations between noise exposure and the risk of dementia. Few large cohort studies have focused on this field, and no high-quality evidence (meta-analysis) has been conducted in the past. To the extent of our knowledge, two systematic reviews have focused on noise exposure and cognitive impairment (14, 28). The two studies were supported by the WHO and delivered as part of the evidence that made up the Environmental Noise Guidelines for the European Region. One of them evaluated the quality of evidence on the effect of environmental noise on cognition, which was based on the population of children (14). Thirty-four eligible studies were included, and most of them were cross-sectional studies. The study found that aircraft noise was associated with poorer long-term memory, reading and oral comprehension in children, with moderate quality of evidence. The study indicated that the lack of evidence from longitudinal and intervention studies was the key limitation of the available study (14). Another study reviewed the associations of environmental noise with mental health, including cognition, dementia and other neurodegenerative outcomes (28). Similar to the study above, the evidence was strong enough to estimate the effects of environmental noise on children's reading comprehension, but the effects on dementia and neurodegenerative results still lacked evidence.

Apart from noise, other environmental factors were concerned with influencing cognitive function. The Lancet Commission published a report about dementia prevention, intervention and care in 2020, in which air pollution was considered a potential risk factor for dementia (54). A systematic review in 2019 similarly pointed toward an association between exposure to pollutants and increased risk of dementia, particularly for PM_2.5_ and NO_x_ (55). Similar to noise, Aβ deposition is considered a potential mechanism of the effect of air pollution on dementia. Some publications have explored the association between air pollution and cognitive function (21, 41, 47, 48, 51). Although most of the studies adjusted the outcome risk factors for air pollution or conducted sensitivity analyses, the results of air pollution and noise may still have cross influence. Furthermore, Tzivian et al. (51) mentioned that the results of noise adjusted by air pollution were more robust than air pollution adjusted by noise. They also concluded that air pollution and traffic noise may synergistically influence cognitive function in adults in another analysis (56). Few studies have focused on the association effect between air pollution and noise. Whether the effect was enhanced or weakened still needs further research wshen air pollution and noise act on cognitive function at the same time.

Nevertheless, some studies have concluded that there was no significant association between noise exposure and dementia risk. Andersson et al. (21) suggested that road traffic noise would not cause dementia and that the effect may be mainly influenced by air pollution exposure. Similar results were found in another study (41). However, both studies considered the effect of air pollution as combined exposure on the outcome. Given that there were great differences in environmental factors and noise values, future studies should be conducted that consider the potential joint effects of multiple environmental exposures.

Although population-based ecological studies or observational studies are limited, some scholars still explore the mechanism of the effect of noise based on animal or cytological experiments. Noise exposure can influence brain structure directly and then damage cognitive function. A study conducted in 1998 reported that exposure to noise stress could significantly damage the cognitive function of the prefrontal cortex by conducting primate experiments (57). In addition, the experiment also suggested that the molecular mechanism of impairment may be associated with a hyperdopaminergic mechanism (57). Similarly, some experimental studies have explored the influence of noise stress and brain structures, indicating that chronic traffic noise can reduce the brain volume in the prefrontal cortex and lead to thickness in the hippocampus and amygdala area, which may activate stress reactions to increase the release of dopamine (58–61). Furthermore, studies have reported that long-term railway noise exposure may damage learning and memory function in the temporal lobe (62, 63). We noticed that several studies mentioned the dopamine release mechanism, indicating that it may be a pathway of the cause of dementia.

Moreover, noise exposure may damage cognitive function via the neuroendocrine pathway. More studies have indicated that noise can activate the hypothalamic–pituitary–adrenal axis (HPA), thus increasing the secretion of adrenocorticotropic hormone, corticosterone and the catecholamine hormone system to affect learning and memory function (24, 64–66). Disorders of the neuroendocrine pathway can result in anxiety-like behavior, learning and memory impairment, and balance dysfunction (59). Aβ peptide deposition and tau protein hyperphosphorylation are considered possible pathological mechanisms of AD (5). Some evidence has also reported that chronic noise exposure may aggravate the deposition of Aβ and tau protein hyperphosphorylation, which might be regulated by the HPA axis (65, 67, 68). Furthermore, glucocorticoids and corticosterone regulated by the HPA axis may affect the oxidative stress response, in turn causing changes in Aβ and tau protein (59, 69).

A number of studies reported similar findings based on their experiments, which lays the foundation for population-based studies in the future. In general, based on previous studies, the mechanism of noise exposure and cognitive impairment is considered to be associated with changes in brain structure, stress reactions, the HPA axis and neuroendocrine factors. As the key link of possible pathways, the HPA axis has been related to other pathways, suggesting that there may be a close connection between several mechanisms.

This study has some limitations. First, although we searched the main common databases more comprehensively, the included studies still lacked quantity, so the number of studies we contained was limited. Second, from the baseline characteristics of the included studies, we found that the noise assessment methods, noise exposure types or outcome assessment methods were different from each other, which may increase the sources of heterogeneity. Limited by the number of included studies, we could not perform further analysis. Third, we included case-control studies and cross-sectional studies, which is not a relatively robust study design to explain the relationship. Compared with cohort studies, case-control studies and cross-sectional studies may likely have recall bias and selection bias, which influence the accuracy of the results. Also we have not investigated the potential sources of heterogeneity formally. However, this is the first quantitative analysis of the relationship between noise exposure and the risk of dementia, providing quantitative evidence for the etiology of dementia. Dose-response meta-analysis further elucidated the relationship of the increment of noise exposure and dementia, which provides accurate data for the prevention and treatment of dementia in the future.

## Conclusion

In conclusion, we found a significant association between noise exposure and the risk of dementia. Based on the current evidence, exposure to noise may be a specific risk factor for dementia. Since current studies mainly focus on community noise and traffic noise, more high-quality longitudinal studies should pay attention to other noise sources, such as occupational noise exposure and risk of dementia. In addition, to better prevent dementia, rigorously designed animal experiments are also needed to explore the etiological mechanism of noise and dementia.

## Data Availability Statement

The original contributions presented in the study are included in the article/Supplementary Material, further inquiries can be directed to the corresponding author/s.

## Author Contributions

LH, LM, and YZ had full access to all of the data in the study, and took responsibility for the integrity of the data and the accuracy of the data analysis and drafted the article. LH, YZ, and SZ designed the study. LH, YZ, and YL developed and tested the data collection forms. LM, YZ, SZ, and FJ acquired the data. LM, YZ, LS, and LH conducted the analysis and interpreted the data. All authors critically revised the article and read and approved the final article.

## Conflict of Interest

The authors declare that the research was conducted in the absence of any commercial or financial relationships that could be construed as a potential conflict of interest.

## Publisher's Note

All claims expressed in this article are solely those of the authors and do not necessarily represent those of their affiliated organizations, or those of the publisher, the editors and the reviewers. Any product that may be evaluated in this article, or claim that may be made by its manufacturer, is not guaranteed or endorsed by the publisher.
