# Supplementary material for: Chronic Noise Exposure and Risk of Dementia: A Systematic Review and Dose-Response Meta-Analysis
Source: Front Public Health. 2022 Jun 20;10:832881. doi: 10.3389/fpubh.2022.832881 (PMC9251202; doi:10.3389/fpubh.2022.832881)
Supplement: Supplementary file 1 [file Table_1.DOCX]

Supplementary Material

# Search Strategy

PubMed

((((Alzheimer Disease [MeSH Terms]) OR (Alzheimer* [ Title/Abstract]) OR (AD [Title/Abstract]) OR (Dement* [ Title/Abstract]) OR (Amentia [Title/Abstract])) OR ((Cognitive Dysfunction [MeSH Terms]) OR (Cognitive Dysfunction [Title/Abstract]) OR (cognitive decline [Title/Abstract]) OR (cognitive impairment [Title/Abstract]) OR (Neurocognitive Disorder [Title/Abstract]) OR (Mental Deterioration [Title/Abstract]) OR (MCI [Title/Abstract]) OR (cognitive disorder [Title/Abstract]))) OR ((Dementia [MeSH Terms]) OR (Deliri* [Title/Abstract]) OR (Lewy* bod* [Title/Abstract]) OR (Huntington* [Title/Abstract]) OR (Pick* disease [Title/Abstract]) OR (Kluver Bucy Syndrome [Title/Abstract]) OR (Creutzfeldt [Title/Abstract]) OR (Chronic cerebrovascular [Title/Abstract]) OR (Wernicke encephalopathy [Title/Abstract]) OR (Subcortical Encephalopathy [Title/Abstract]) OR (Subcortical Leukoencephalopathy [Title/Abstract]) OR (Mesulam* Syndrome [Title/Abstract]) OR (Organic brain disease [Title/Abstract]) OR (supranuclear palsy [Title/Abstract]) OR (Cerebr* insufficien*[Title/Abstract]) OR (Frontotemporal Lobar Degeneration [Title/Abstract]) OR (FTD [Title/Abstract]) OR (FTLD [Title/Abstract]) OR (Binswanger*[Title/Abstract]) OR (benign senescent forgetfulness[Title/Abstract]))) AND ((Noise [MeSH Terms]) OR (noise [Text Word]) OR (sound [Text Word]))

EMBASE via OVID

#1 exp Alzheimer disease/

#2 Alzheimer*. ab

#3 AD. ab

#4 Dement*. ab

#5 diffuse cortical sclerosis. ab

#6 #1 OR #2 OR #3 OR #4 OR #5

#7 exp cognitive defect/

#8 cognitive defect. ab

#9 cognitive deficit. ab

#10 cognition disorder. ab

#11 cognition defect. ab

#12 cognitive disability. ab

#13 cognitive disorder. ab

#14 cognitive dysfunction. ab

#15 cognitive impairment. ab

#16 MCI. ab

#17 delirium. ab

#18 #7 OR #8 OR #9 OR #10 OR #11 OR #12 OR #13 OR #14 OR #15 OR #16 OR #17

#19 exp dementia/

#20 dement*. ab

#21 amentia. ab

#22 intellectual impairment. ab

#23 CADASIL. ab

#24 Lewy* bod*. ab

#25 Huntington*. ab

#26 Kluver Bucy. ab

#27 mental deterioration. ab

#28 neuronal ceroid lipofuscinosis. ab

#29 pick* disease. ab

#30 rett syndrome. ab

#31 tauopathy. ab

#32 frontotemporal lobar degeneration. ab

#33 FTD. ab

#34 FTLD. ab

#35 #19 OR #20 OR #21 OR #22 OR #23 OR #24 OR #25 OR #26 OR #27 OR #28 OR #29 OR #30 OR #31 OR #32 OR #33 OR #34

#36 exp noise/

#37 exp noise pollution/

#38 noise*.tw

#39 sound*.tw

#40 noisy environment.tw

#41 sound pollution.tw

#42 #36 OR #37 OR #38 OR #39 OR #40 OR #41

Cochrane Library via OVID

#1 exp Alzheimer disease/

#2 Alzheimer*. ab

#3 AD. ab

#4 Dement*. ab

#5 diffuse cortical sclerosis. ab

#6 #1 OR #2 OR #3 OR #4 OR #5

#7 exp cognitive defect/

#8 cognitive defect. ab

#9 cognitive deficit. ab

#10 cognition disorder. ab

#11 cognition defect. ab

#12 cognitive disability. ab

#13 cognitive disorder. ab

#14 cognitive dysfunction. ab

#15 cognitive impairment. ab

#16 MCI. ab

#17 delirium. ab

#18 #7 OR #8 OR #9 OR #10 OR #11 OR #12 OR #13 OR #14 OR #15 OR #16 OR #17

#19 exp dementia/

#20 dement*. ab

#21 amentia. ab

#22 intellectual impairment. ab

#23 CADASIL. ab

#24 Lewy* bod*. ab

#25 Huntington*. ab

#26 Kluver Bucy. ab

#27 mental deterioration. ab

#28 neuronal ceroid lipofuscinosis. ab

#29 pick* disease. ab

#30 rett syndrome. ab

#31 tauopathy. ab

#32 frontotemporal lobar degeneration. ab

#33 FTD. ab

#34 FTLD. ab

#35 #19 OR #20 OR #21 OR #22 OR #23 OR #24 OR #25 OR #26 OR #27 OR #28 OR #29 OR #30 OR #31 OR #32 OR #33 OR #34

#36 exp noise/

#37 exp noise pollution/

#38 noise*.tw

#39 sound*.tw

#40 noisy environment.tw

#41 sound pollution.tw

#42 #36 OR #37 OR #38 OR #39 OR #40 OR #41

# Supplementary Table S1. The NOS scale of the included cohort studies

| First author, year | NOS | Selection | | | | Comparability | Outcome | | |
| --- | --- | --- | --- | --- | --- | --- | --- | --- | --- |
|  |  | Representativeness of the exposed cohort | Selection of the nonexposed cohort | Ascertainment of exposure | Demonstration that outcome of interest was not present at start of study | Comparability of cohorts on the basis of the design or analysis | Assessment of outcome | Was follow-up long enough for outcomes to occur | Adequacy of follow up of cohorts |
| Andersson, 2018 | 7 | 1 | 1 | 1 | 1 | 1 | 1 | 1 | 0 |
| Tyas, 2001 | 7 | 1 | 1 | 1 | 1 | 1 | 0 | 1 | 1 |
| Weuve, 2020 | 8 | 1 | 1 | 1 | 1 | 2 | 1 | 1 | 0 |
| Yuchi, 2020 (b) | 9 | 1 | 1 | 1 | 1 | 2 | 1 | 1 | 1 |
| Fuks, 2019 | 7 | 0 | 1 | 1 | 0 | 2 | 1 | 1 | 1 |
| Yu, 2020 | 7 | 0 | 1 | 1 | 0 | 2 | 1 | 1 | 1 |
| Carey,2018 | 8 | 1 | 1 | 1 | 1 | 2 | 1 | 1 | 0 |
| Cantuaria, 2021 | 8 | 1 | 1 | 1 | 1 | 2 | 1 | 1 | 0 |

# Supplementary Table S2. The NOS scale of the included case-control study

| First author, year | NOS | Selection | | | | Comparability | Exposure | | |
| --- | --- | --- | --- | --- | --- | --- | --- | --- | --- |
|  |  | Case definition adequacy | Representativeness of the cases | Selection of Controls | Definition of Controls | Comparability of cases and controls on the basis of the design or analysis | Ascertainment of exposure | Same method of ascertainment for cases and controls | Non-Response rate |
| Yuchi, 2020 (a) | 7 | 1 | 1 | 1 | 0 | 1 | 1 | 1 | 1 |

# Supplementary Table S3. The AHRQ scale of the included cross-sectional studies

| First author, year | AHRQ | 1 | 2 | 3 | 4 | 5 | 6 | 7 | 8 | 9 | 10 | 11 |
| --- | --- | --- | --- | --- | --- | --- | --- | --- | --- | --- | --- | --- |
| Tzivian, 2016 | 10 | 1 | 1 | 1 | 1 | 1 | 1 | 1 | 1 | 0 | 1 | 1 |
| Crous, 2020 | 6 | 1 | 1 | 1 | 1 | 1 | 0 | 0 | 1 | 0 | 0 | 0 |

The 11 assessment terms of AHRQ were: 1 Define the source of information (survey, record review); 2 List inclusion and exclusion criteria for exposed and unexposed subjects (cases and controls) or refer to previous publications; 3 Indicate time period used for identifying patients; 4 Indicate whether or not subjects were consecutive if not population-based; 5 Indicate if evaluators of subjective components of study were masked to other aspects of the status of the participants; 6 Describe any assessments undertaken for quality assurance purposes (e.g., test/retest of primary outcome measurements); 7 Explain any patient exclusions from analysis; 8 Describe how confounding was assessed and/or controlled; 9 If applicable, explain how missing data were handled in the analysis; 10 Summarize patient response rates and completeness of data collection; 11 Clarify what follow-up, if any, was expected and the percentage of patients for which incomplete data or follow-up was obtained.

# Supplementary Table S4. The risk estimate (95% CI) between noise exposure and dementia outcomes from each reviewed study

| First author | Dementia | AD | VaD/NAD | MCI/CIND | Adjustment variables |
| --- | --- | --- | --- | --- | --- |
| Andersson, 2018 | 0.99 (0.62, 1.59) | - | - | - | Baseline age |
|  | 0.97 (0.58, 1.60) | - | - | - | Baseline age, education, physical activity, smoking, sex, body mass index, waist–hip ratio, alcohol, and ApoE4 |
|  | 0.95 (0.57, 1.57) | - | - | - | Baseline age, education, physical activity, smoking, sex, body mass index, waist–hip ratio, alcohol, ApoE4, baseline medical history of diabetes, hypertension, and stroke |
| Weuve, 2020 | - | 1.29 (1.08, 1.55) | - | 1.36 (1.15, 1.62) | Calendar time, baseline age, age at exam, sex, race/ethnicity, income, education, neighborhood SES, smoking, alcohol use, and air pollution (NOx) |
| Yuchi, 2020 | - | 0.99 (0.92, 1.08) | 1.01 (0.99, 1.04) | - | Comorbidities, household income, education and ethnicity (AD)/Age, sex, comorbidities, household income, education and ethnicity (NAD) |
| Fuks, 2019 | - | - | - | 1.69 (0.94, 3.04) (Residental, Lden) | Age, smoking, passive smoking, educational level |
|  | - | - | - | 0.87 (0.51, 1.49) (Residental, Lnight) | Age, smoking, passive smoking, educational level |
|  | - | - | - | 0.68 (0.41, 1.14) (Annoyance, day) | Age, smoking, passive smoking, educational level |
|  | - | - | - | 0.64 (0.35, 1.17) (Annoyance, night) | Age, smoking, passive smoking, educational level |
|  | - | - | - | 1.84 (1.01, 3.38) (Residental, Lden) | Age, smoking, passive smoking, educational level and noise |
|  | - | - | - | 0.95 (0.54, 1.65) (Residental, Lnight) | Age, smoking, passive smoking, educational level and noise |
|  | - | - | - | 0.62 (0.36, 1.04) (Annoyance, day) | Age, smoking, passive smoking, educational level and noise |
|  | - | - | - | 0.65 (0.35, 1.21) (Annoyance, night) | Age, smoking, passive smoking, educational level and noise |
|  | - | - | - | 1.87 (0.99, 3.52) (Residental, Lden) | Age, smoking, passive smoking, educational level and PM10 and NO2 |
|  | - | - | - | 0.83 (0.47, 1.48) (Residental, Lnight) | Age, smoking, passive smoking, educational level and PM10 and NO2 |
|  | - | - | - | 0.63 (0.37, 1.07) (Annoyance, day) | Age, smoking, passive smoking, educational level and PM10 and NO2 |
|  | - | - | - | 0.58 (0.31, 1.09) (Annoyance, night) | Age, smoking, passive smoking, educational level and PM10 and NO2 |
|  | - | - | - | 1.68 (0.93, 3.04) (Residental, Lden) | Age, smoking, passive smoking, educational level and depression |
|  | - | - | - | 0.87 (0.51, 1.49) (Residental, Lnight) | Age, smoking, passive smoking, educational level and depression |
|  | - | - | - | 0.67 (0.40, 1.13) (Annoyance, day) | Age, smoking, passive smoking, educational level and depression |
|  | - | - | - | 0.63 (0.34, 1.16) (Annoyance, night) | Age, smoking, passive smoking, educational level and depression |
| Yu, 2020 | 1.2 (0.93, 1.5) | - | - | - | Baseline age, gender, years of education and traffic-related NOx |
|  | 1.2 (0.97, 1.6) | - | - | - | Baseline age, gender, years of education, occupation during most of  life, smoking status, alcohol consumption status, physical activity level, NSES indicator,  residential county, baseline Charlson index, baseline cognition function, and primary  Baseline age, gender, years of education, occupation during most of life, smoking status, alcohol consumption status, physical activity level, NSES indicator residential county, baseline Charlson index, baseline cognition function, primary language and traffic-related NOx |
| Yu, 2020 (b) | 1.7 (0.89, 3.1) (obesity) | - | - | - | Baseline age, sex, education, occupation held during most of the life, NSES, smoking status, alcohol status, residential county, physical activity and household income, and baseline cognition function |
|  | 2.2 (1.7, 3.9) (hyperglycemia) | - | - | - | Baseline age, sex, education, occupation held during most of the life, NSES, smoking status, alcohol status, residential county, physical activity and household income, and baseline cognition function |
|  | 1.8 (1.0, 3.0) (low HDL-cholesterol) | - | - | - | Baseline age, sex, education, occupation held during most of the life, NSES, smoking status, alcohol status, residential county, physical activity and household income, and baseline cognition function |
| Tzivian, 2016 | - | - | - | 1.40 (1.03, 1.91) (Lden) | Age, sex, socioeconomic status, alcohol consumption, smoking status, self-reported environmental tobacco smoke, any regular physical activity, body mass index |
|  | - | - | - | 1.80 (1.07, 3.04) (Lnight) | Age, sex, socioeconomic status, alcohol consumption, smoking status, self-reported environmental tobacco smoke, any regular physical activity, body mass index |
| Carey, 2018 | - | 1.03 (0.99, 1.07) | 1.00 (0.96, 1.05) | - | Age, sex, ethnicity, smoking, alcohol consumption, body mass index and Index of Multiple Deprivation |
| Cantuaria, 2021 | - | 1.16 (1.11, 1.22) (Lden max≥65, road) | 1.19 (1.09, 1.31) (Lden max≥65, road) | - | Age (by design), sex, calendar year, civil status, income, region of origin, occupational status, proportion of high quality green space, a number of area level, percentage population with low income, only basic education, unemployment, manual labour occupation, single parent, and a criminal record |
|  | - | 1.27 (1.22, 1.34) (Lden min≥55, road) | 1.14 (1.04, 1.25) (Lden min≥55, road) | - | Age (by design), sex, calendar year, civil status, income, region of origin, occupational status, proportion of high quality green space, a number of area level, percentage population with low income, only basic education, unemployment, manual labour occupation, single parent, and a criminal record |
|  | - | 1.16 (1.10, 1.23) (Lden max≥60, railway) | 1.09 (0.97, 1.22) (Lden max≥60, railway) | - | Age (by design), sex, calendar year, civil status, income, region of origin, occupational status, proportion of high quality green space, a number of area level, percentage population with low income, only basic education, unemployment, manual labour occupation, single parent, and a criminal record |
|  | - | 1.24 (1.17, 1.30) (Lden min≥50, railway) | 1.03 (0.93, 1.14) (Lden min≥50, railway) | - | Age (by design), sex, calendar year, civil status, income, region of origin, occupational status, proportion of high quality green space, a number of area level, percentage population with low income, only basic education, unemployment, manual labour occupation, single parent, and a criminal record |

-: Not reported.
